# Use of hamster as a model to study diet-induced atherosclerosis

**DOI:** 10.1186/1743-7075-7-89

**Published:** 2010-12-10

**Authors:** Alice Dillard, Nirupa R Matthan, Alice H Lichtenstein

**Affiliations:** 1 Cardiovascular Nutrition Laboratory, Jean Mayer U.S. Department of Agriculture, Human Nutrition Research Center on Aging at Tufts University, 711 Washington Street, Boston, MA 02111, USA

## Abstract

Golden-Syrian hamsters have been used as an animal model to assess diet-induced atherosclerosis since the early 1980s. Advantages appeared to include a low rate of endogenous cholesterol synthesis, receptor-mediated uptake of LDL cholesterol, cholesteryl ester transfer protein activity, hepatic apoB-100 and intestinal apoB-48 secretion, and uptake of the majority of LDL cholesterol via the LDL receptor pathway. Early work suggested hamsters fed high cholesterol and saturated fat diets responded similarly to humans in terms of lipoprotein metabolism and aortic lesion morphology. Recent work has not consistently replicated these findings. Reviewed was the literature related to controlled hamster feeding studies that assessed the effect of strain, background diet (non-purified, semi-purified) and dietary perturbation (cholesterol and/or fat) on plasma lipoprotein profiles and atherosclerotic lesion formation. F1B hamsters fed a non-purified cholesterol/fat-supplemented diet had more atherogenic lipoprotein profiles (nHDL-C > HDL-C) than other hamster strains or hamsters fed cholesterol/fat-supplemented semi-purified diets. However, fat type; saturated (SFA), monounsaturated or n-6 polyunsaturated (PUFA) had less of an effect on plasma lipoprotein concentrations. Cholesterol- and fish oil-supplemented semi-purified diets yielded highly variable results when compared to SFA or n-6 PUFA, which were antithetical to responses observed in humans. Dietary cholesterol and fat resulted in inconsistent effects on aortic lipid accumulation. No hamster strain was reported to consistently develop lesions regardless of background diet, dietary cholesterol or dietary fat type amount. In conclusion, at this time the Golden-Syrian hamster does not appear to be a useful model to determine the mechanism(s) of diet-induced development of atherosclerotic lesions.

## Introduction

Cardiovascular disease (CVD) is the leading cause of mortality in developed countries and is a growing health challenge in developing countries [1,2]. The majority of CVD is attributed to atherosclerosis, characterized by endothelial dysfunction, chronic inflammation, dyslipidemia, and accumulation of lipid in arterial walls [1,3-10]. Data from both observational and interventional studies indicate that dietary modification can alter atherosclerotic lesion progression [5,11,12]. Although the diet/CVD relationship was first identified at the turn of the 20^th ^century [13], salient issues related to dietary fat type are still in question [14-16].

Randomized controlled human intervention trials in the field of diet and CVD are rare, in part due to the complexity and cost of executing the studies, limited number of validated surrogate biomarkers to track disease progression, and inaccessibility of pivotal tissues/organs necessary to determine underlying mechanisms. The availability of an animal model addresses the later issue by allowing the assessment of diet and atherosclerosis development in multiple tissue systems simultaneously. This in turn facilitates a more complete understanding of the complex relationship between diet and CVD risk. In general, unmodified rats and mice are not suitable animal models to study diet-induced changes in plasma lipid and lipoprotein concentrations and atherosclerotic lesion development because they do not develop aortic lesions or an atherogenic lipoprotein profile [non-high-density lipoprotein cholesterol (nHDL-C) > high-density lipoprotein cholesterol (HDL-C)] similar to that observed in humans. Transgenic, knock-out, and knock-down mouse models have been used successfully to study discrete components of the system [17-21], but it is difficult to use these models to assess multi-component etiologies. Such questions are best investigated using unmodified animal models.

Since the 1980s hamsters have been used as an animal model to assess diet-induced atherosclerosis [22]. Relative to other unmodified rodent models, the hamster was thought to be preferable due to its apparent low rate of endogenous cholesterol synthesis, receptor-mediated uptake of low density lipoprotein cholesterol, presence of cholesteryl ester transfer protein (CETP) activity [23-28], secretion of apolipoprotein (apo) B-100 from the liver and apo B-48 from the small intestine [29], and uptake of the majority of LDL-C via the LDL receptor pathway [22]. The morphology of aortic foam cells and lesions in hamsters fed atherogenic diets was reported to be similar to human lesions [22,30,31].

More recent work has not consistently replicated the plasma lipoprotein response or aortic lesion morphology in hamsters that was previously shown to be similar to humans [31-43]. Our aim was to review the literature regarding diet interventions in Golden-Syrian hamsters, and plasma lipid and lipoprotein response and aortic lesion formation.

## Methods

### Literature Search Strategy and Data Extraction

A literature search was conducted through January, 2010 in PubMed. The following search terms were used: dietary cholesterol, dietary fat, and fatty acids. These terms were crossed with hamster. Then the terms atherosclerosis and plasma lipoprotein were searched independently and then crossed with hamster. Finally, the results from the two searches were crossed. The search was limited to English language publications. To ensure completeness this method was supplemented by reviewing citations in recent publications. Extracted data included intervention period, male hamster strain, male hamster age, background diet, and diet composition. For the later point emphasis was placed on dietary cholesterol amount, and dietary fat type and amount.

## Results

### Outcome Measures

#### Plasma lipid and lipoproteins

Studies that reported plasma cholesterol (total cholesterol, HDL-C and nHDL-C) and/or plasma triglyceride (TG) concentrations were included in the data summaries. All plasma lipids were measured after hamsters were food deprived.

#### Aortic lesion development

In general, two approaches have been used to measure aortic lesions. The first method was oil red-O lipid staining. These data were expressed using multiple systems; μm^2 ^fatty streak/mm^2 ^aorta [31,35,36,44-46], percent total aortic lesion area [47,48], percent aortic arch fatty streak area [49-51], percent foam cell area [52] or aortic cross-sections as percent total "internal elastic lamina" [30]. The use of multiple reporting systems made cross comparisons among studies challenging. The second method was determining aortic cholesteryl ester (CE) content. The data tended to be either expressed as either CE/mg aortic wet weight [37,39,53-57] or dry weight [58]. Oil red-O staining provided a measure of lesion surface area, but no data on the nature and invasiveness of the lesion. CE content provided an assessment of total aortic cholesterol accumulation, but did not provide an indication of the lesion type.

### Covariates

Intervention period, hamster strain and sex, background diet and diet composition, including amount of cholesterol and type and amount of fat, were found to affect outcome measures.

#### Intervention Periods

Intervention periods ranged from 3 weeks [59,60] to 12 months [22,61]. The most common intervention periods ranged from 10 and 12 weeks [33-35,37,42,43,46,48,51,53-56,62-66]. Outcome measures for the shorter term studies (< 6 weeks) were primarily plasma lipoprotein profiles and for the mid- to long-term studies (6-12 weeks) was aortic lesion formation.

#### Hamster Strain

Four Golden-Syrian hamster strains have been predominantly used to study diet-induced atherosclerosis. These strains can be characterized as being derived from inbred or outbred hamsters. In common usage, the one inbred strain was the F1B hamster from Biobreeders (Watertown, MA). The three outbred strains were Charles River (CR) (Wilmington, MA), Sasco (Omaha, NE) and Harlan (Indianapolis, IN).

#### Hamster Sex

The vast majority of studies used male hamsters only (115 out of 120). Seven studies included female hamsters [38,43,67-71]. Of those, three studies reported on fetal cholesterol metabolism only [69-71], two studies compared plasma lipoprotein concentrations or atherosclerotic lesion development between female ovariectomized and sham-operated hamsters [67,68], and two studies compared plasma lipoprotein concentrations and atherosclerotic lesion development between male and female hamsters [38,43]. Due to the limited amount of data and lack of comparable data, outcome measures for female hamsters were not included.

#### Background Diet

Two distinct types of background diets have been used for hamster studies: semi-purified and non-refined. Semi-purified diets were composed of refined ingredients such as casein and sucrose, whereas non-purified diets were composed of unmodified ingredients such as grains and grain products. A standard vitamin and mineral mix was added to both [72]. Semi-purified diets were generally favored because they provide more uniformity and reproducibility.

#### Diet Composition

##### Dietary Cholesterol

Studies designed to asses diet-induced changes in plasma lipids or atherosclerosis, for the most part, include supplemental cholesterol. The levels used in most hamster studies exceed what was considered high intake in humans. The amount of cholesterol was of concern because large amounts (≥1% w/w) were hepatotoxic; impairing normal lipoprotein metabolism [30,55,73]. Hepatotoxicity has been characterized by visual inspection of the liver or measuring hepatic cholesterol content, hepatic lipoprotein metabolism and apolipoprotein synthesis [23,25,55]. The amount of supplemental dietary cholesterol identified ranged from 0 to 3% (w/w) [22,30,64,74]. The majority of studies have used between 0.05 to 0.2% cholesterol (w/w) [30-36,39,40,42,43,46-48,51-56,59,62,63,66,75-94].

##### Dietary Fat

Studies feeding hamsters diets supplemented fat were examined to determine if fatty acid chain length and degree of unsaturation alter plasma lipids or aortic lesion development.

###### Dietary Fat Type

The amount of supplemental dietary fat has ranged from 0 to 20% (w/w) [30,32,36,48,51,62,76-78,85,90,95-97], with 10% (w/w) used most frequently [31,33-35,37-43,45,47,50,52-56,59,63,65,66,79-84,88,92,94,98,99]. Ten percent saturated fatty acids (SFA) with supplemental cholesterol has been consistently reported to raise plasma total cholesterol and nHDL-C concentrations [31,38,47,50,55,62] without having adverse effects on hepatic function [51]. The most commonly used sources of SFA were coconut oil [31-35,39-44,47,50,52,54,56,59,61,63,66,80,83,84,86,88,94,99,100], fully-hydrogenated coconut oil [32,38,48,62,78,82] and butter [22,30,47,49,51,53,55,57,58,85,101,102]. Comparison fats were either monounsaturated fatty acids (MUFA) or omega-6 polyunsaturated fatty acids (n-6 PUFA). The most commonly used source of MUFA were canola [44,47,53] and olive [37,56,76,98] oils, and n-6 PUFA were soybean [47,53,94], safflower [56,102] and sunflower [37,45,46] oils. The most commonly used source of very long chain omega-3 PUFA (n-3 PUFA) was fish oil [95,96,102-104].

### Data Presentation

Due to the nature of the data identified it was presented within subsections; plasma lipids and lipoproteins, and aortic lesion. Within each subsection, the data were presented by hamster strain, background diet, dietary cholesterol and dietary fat type. The figures are a visual representation summarizing the data extracted. They are one approach to represent the range of findings. Observations discussed in the plasma lipid and lipoprotein and aortic lesion subsections were made on the basis of visual assessments rather than statistical analysis which was precluded due to the heterogeneous nature of the data in terms of study designs, dietary perturbations and hamster strains.

### Plasma Lipid and Lipoproteins

#### Hamster Strain

**Figure 1:**
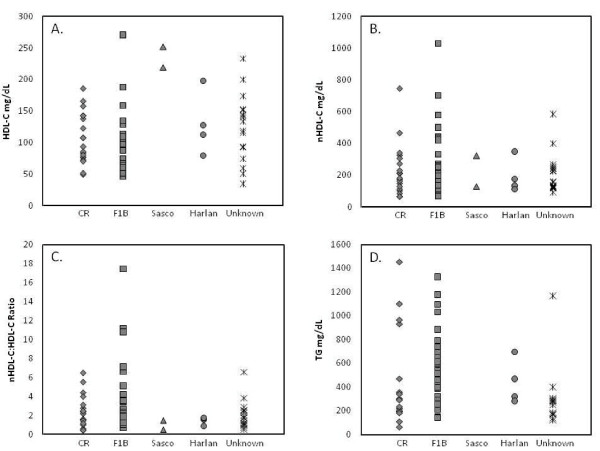
**Plasma lipoprotein concentrations in Golden-Syrian hamsters fed cholesterol- and SFA-supplemented non-purified and semi-purified diets**. Plasma (A) HDL-C, (B) nHDL, (C) nHDL-C:HDL ratio and (D) TG concentrations in different strains of the Golden-Syrian hamster fed cholesterol- and SFA-supplemented diets.

In response to cholesterol- and SFA-supplemented diets, the inbred F1B [28,31-33,35,39,41-44,54-56,59,61-63,77,80,86,94,96,98,100,105] hamster has been more consistently reported to develop an atherogenic lipoprotein profile (nHDL-C > HDL-C) than any of the three outbred strains, CR [28,34,36,40,47-49,53,55,66,84,88,94,99,102,106], Sasco [30,51] and Harlan [28,85,107] hamsters (Figure 1A-C). In the outbred strains, in contrast to that normally observed in humans, the HDL-C concentrations were equal to or slightly higher than nHDL-C concentrations. Noteworthy was the wide range of plasma cholesterol concentrations after the hamsters were fed a cholesterol- and SFA-supplemented diet, regardless of strain (Figure 1A-C) [22,24,28,30-36,38-44,47-59,61-64,66,75,77,78,80-86,88,89,92,94,96,98-100,105-114]

Inbred and outbred hamster strains exhibited a wide range of plasma TG concentrations, with the F1B hamsters exhibiting the widest absolute range (119 to 1350 mg/dL) [28,31,33,35,38,39,41-44,52,54-56,59,62,63,77,80,86,94,96,98,100,105] (Figure 1D). The hypertriglyceridemia in the inbred and outbred hamster strains fed cholesterol- and SFA-supplemented diet appeared to be inconsistent with the response in humans, which tended to be null or increased slightly [115,116].

Age at initiation of dietary intervention did not show a clear pattern with regard to plasma lipoprotein concentrations and ranged from 2 to 28 weeks. Of note, over half of F1B hamsters started a study at 8 weeks of age. Amount of dietary cholesterol and amount and type of dietary fat could partially explain the wide range of plasma lipoprotein concentrations reported and will be discussed in subsequent sections. Because the majority of data available relate to the F1B hamster the subsequent discussion will be limited to that strain; however, data for all the strains was summarized in supplementary tables and figures.

#### Background Diet

**Figure 2:**
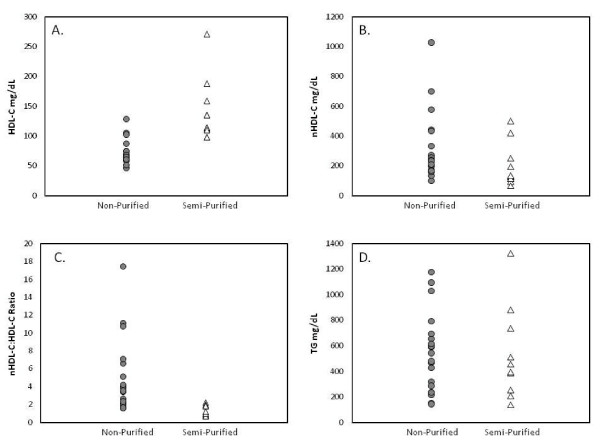
**Plasma lipoprotein concentrations in F1B hamsters fed cholesterol- and SFA-supplemented non-purified or semi-purified diets**. Plasma (A) HDL-C, (B) nHDL, (C) nHDL-C:HDL ratio and (D) TG concentrations in F1B hamsters fed cholesterol- and SFA-supplemented non-purified or semi-purified diets.

F1B hamsters fed non-purified diets supplemented with cholesterol and SFA [22,31,33-36,38-44,47,48,50,52,53,55,58,59,61,63,64,66,80,82,84-86,88,98-100,105-107,114] resulted in lower HDL-C (Figure 2A) and higher nHDL-C concentrations (Figure 2B) than the same strain fed a semi-purified diet supplemented with cholesterol and SFA [30,32,46,51,54,56,62,75,77,78,81,83,89,92,94,102,108-110,112,113]. Of note, the resulting nHDL-C:HDL-C ratios were higher in the majority of studies using non-purified, but not semi-purified diets (Figure 2C). No notable difference in response to the two background diets in plasma TG concentrations was observed (Figure 2D) [31,33-49,52-56,59,62,63,66,76,77,80,84-86,88,94-96,98,100-102,107].

With respect to differences identified in plasma lipoprotein concentrations resulting from the two background diets, potential differences in fiber and/or other non-essential dietary components, such as phytochemicals, between the non-purified and semi-purified diets could account for the differences. No data addressing this variable was identified.

#### Diet Composition

##### Dietary Cholesterol

In the absence of supplemental dietary fat, the higher the dietary cholesterol, the higher the resulting nHDL-C concentrations [53,73,87,117,118]. Hamsters fed a non-purified diet supplemented with 1% (w/w) cholesterol resulted in higher nHDL-C concentrations [117] compared to hamsters fed similar diets containing less cholesterol [0.1-0.2% (w/w)] [53,73,87,118]. Cholesterol added to semi-purified diets ranging from 0.1-0.3% (w/w) without supplemental fat resulted in similar nHDL-C concentrations [79,82,91,119,120]. There were no studies in hamsters fed semi-purified diets with greater than 0.3% (w/w) cholesterol in the absence of supplemental fat.

Compared to cholesterol-supplemented semi-purified diets [79,82,91,119], supplementing non-purified diets [53,87,117,118] with cholesterol, in the absence of supplemental fat, was reported to result in similar nHDL-C concentrations (Additional file 1**Figure S1B**). Noteworthy, cholesterol-supplemented semi-purified diets [79,82,91,119] without supplemental fat consistently resulted in higher HDL-C concentrations compared to non-purified diets [53,87,117,118] (Additional file 1**Figure S1A**).

#### Dietary Fat

##### SFA, MUFA and n-6 PUFA

**Figure 3:**
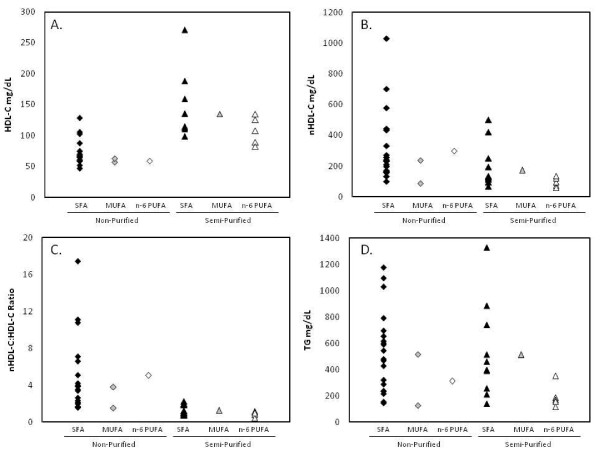
**Plasma lipoprotein concentrations in F1B hamsters fed cholesterol- and SFA-, MUFA, or n-6 PUFA-supplemented diets**. Plasma (A) HDL-C, (B) nHDL, (C) nHDL-C:HDL ratio and (D) TG concentrations in F1B hamsters fed cholesterol- and SFA-, MUFA- or n-6 PUFA-supplemented non-purified or semi-purified diets.

In response to cholesterol- and SFA-, MUFA- or n-6 PUFA-supplemented diets, HDL-C concentrations were higher, nHDL-C:HDL-C ratio were lower, and nHDL-C and TG concentrations were similar in F1B hamsters fed semi-purified diets compared to non-purified diets (Figure 3A-D) [28,44,46,56,94,98,105]. However, there were no differences in plasma lipoprotein concentrations among fat types in hamsters fed either non-purified or semi-purified diets (Figure 3A-D).

Of note was the trend of a positive relationship between HDL-C and TG concentrations in F1B hamsters fed semi-purified diets regardless of fat type (Figure 3A&3D). This response was in contrast to humans, who tend to exhibit an inverse relationship between the two lipoprotein fractions [121].

##### Very Long Chain n-3 PUFA

**Figure 4:**
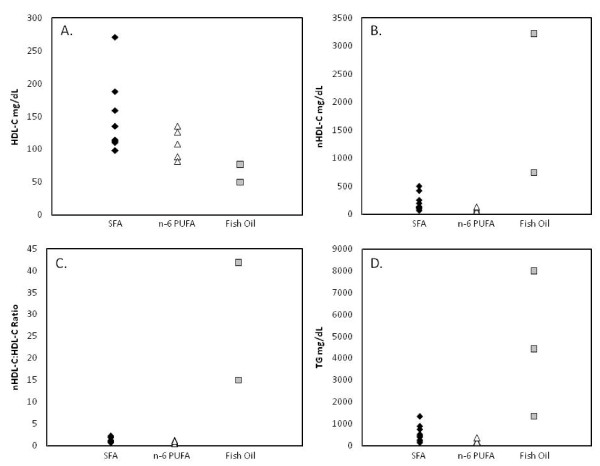
**Plasma lipoprotein concentrations in F1B hamsters fed cholesterol- and SFA-, n-6PUFA- or fish oil-supplemented semi-purified diets**. Plasma (A) HDL-C, (B) nHDL, (C) nHDL-C:HDL ratio and (D) TG concentrations in F1B hamsters fed cholesterol- and SFA-, n-6 PUFA- or fish oil-supplemented non-purified or semi-purified diets.

Notable was an anomaly in the F1B hamster with regard to fish oil (very long chain n-3 fatty acid) and plasma lipoprotein response. In humans, fish oil has little effect on plasma cholesterol concentrations [122]. Similarly, in F1B hamsters fed fish oil-supplemented semi-purified diets without cholesterol there was little effect on plasma cholesterol concentrations (data not shown) [96,104]. However, when cholesterol was included in the diet, the response was highly variable and in some cases resulted in extreme elevations in nHDL-C concentrations (Figure 4A-C) [56,96,102,104]. Similar data using non-purified diets supplemented with fish oil were unavailable.

In hypertriglyceridemic patients, high intakes (3-5 grams/day) of very long chain n-3 fatty acids decreased plasma TG concentrations [123]. In contrast, hamsters fed cholesterol- and fish oil-supplemented semi-purified diets plasma TG concentrations increased 1- to 5-fold compared to cholesterol- and SFA- or n-6 PUFA-supplemented semi-purified diets (Figure 4D) [95,96,103]. When data were available for other hamster strains this trend was not observed (Additional file 2**Figure S2**).

### Aortic Lesion

#### Hamster Strain

In response to cholesterol- and SFA-supplemented diets, three studies reported that F1B [31,38,100] and CR [22,49,55] and one unknown strain of hamster [50] responded with an increase in lesion lipid or foam cell formation compared to chow fed hamsters (Table 1). In contrast, one study in F1B [55], one study in CR [47] and one in an unknown strain [58] reported no lesion development compared to chow (Table 1). The only aortic lesion development data reported for Sasco hamsters compared diets supplemented with cholesterol and SFA, with no comparison to chow or an unsupplemented diet [30,51]. No studies were identified that reported data on the development of aortic lesions in Harlan hamsters. The method used to characterize aortic lesion development, age at initiation of dietary intervention, and length of dietary intervention was different among studies, making cross comparisons difficult to interpret (Table 1).

**Table 1:** Golden-Syrian hamsters fed cholesterol and SFA-supplemented non-purified diets compared to chow.

								Chow	mg/dL			SFA	mg/dL		
								
Ref	Strain	Age (weeks)	Study Length (weeks)	% Fat (w/w)	% CH (w/w)	HDL-C	nHDL-C	nHDL-C:HDL-C	TG	HDL-C	nHDL-C	nHDL-C:HDL-C	TG	Lesion	Lesion Measure
[38]	F1B	8	5	10^1^	0.05				150				650	↑	Aorta stained (μ^2^)

[31]	F1B	8	8	10^2^	0.05	58	58	1.0	87	88	234	2.7	216	↑	Foam cells/mm^2^ aorta

[55]	F1B	8	12	10^3^	0.1	48	35	0.7	110	67	444	6.6	545	↔	CE (ug)/aorta (mg wet weight)

[55]	CR	8	12	10^3^	0.1	68	67	1.0	129	86	210	2.4	195	↑	CE (ug)/aorta (mg wet weight)

[49]	CR	10	24	5^3^	2	50	47	0.9	186	247	581	2.4	1912	↑	Fatty streak area (%)

[47]	CR	8	6	10^2^	0.1	71	25	0.4	219	108	112	1.0	341	↔	Aorta with lesion (% )

[47]	CR	8	6	10^3^	0.1	71	25	0.4	219	108	174	1.6	301	↔	Aorta with lesion (% )

[58]	UNK	3-4	28	5^3^	1				120				169	↔	EC (ug)/aorta (10mg dry weight)

[50]	UNK	5	40	10^2^	0.3	87	64	0.7	102	153	224	1.5	295	↑	Fatty streak area (%)

SFA: saturated fatty acid; CH: cholesterol; HDL-C: high-density lipoprotein cholesterol; nHDL-C: non-HDL-C; TG: triglycerides; CR: Charles River; UNK: unknown hamster strain; EC: esterified cholesterol. SFA source: ^1^Hydrogenated coconut oil, ^2^Coconut oil and ^3^Butter

### Background Diet

Despite differences in the plasma lipoprotein profile, the dearth of data precluded an assessment of whether background diet onto which the cholesterol and SFA was superimposed altered aortic lesion development.

#### Diet Composition

##### Dietary Cholesterol

Only two studies were identified that reported the effect of dietary cholesterol on aortic lesion development. Dietary cholesterol at 0.12 and 0.2% (w/w) added to a semi-purified diet in the absence of supplemental fat increased stained aortic lesion area compared to a non-supplemented semi-purified diet [46,91]. Similar data using non-purified diets were unavailable.

As noted with the plasma lipoprotein response, a wide range in degree of aortic lesion development was noted after the hamsters were fed a cholesterol- and SFA-supplemented diet, regardless of strain (Table 1) [31,38,47,49,50,55,58].

##### Dietary Fat

Five studies were identified that contained data on aortic lesion development in F1B hamsters in response to different types of dietary fat. Regardless of background diet or supplemental cholesterol, no differences were observed in response to dietary fat type [39,54,56,105] (Table 2). The multiple methods used to assess aortic lesion formation did not appear to impact the outcomes reported. Considering the data from all the hamster strains simultaneously did not alter this conclusion (Additional file 3: **Table S1**). However, 2 out of 8 studies reported a larger fatty streak area in cholesterol- and SFA-supplemented non-purified diets compared to cholesterol- and MUFA-supplemented non-purified diets [36,64]. When comparing stained fatty streak area between cholesterol- and MUFA- or n-6 PUFA-supplemented hamsters, the latter group exhibited more lesion [45] and aortic CE accumulation [37].

**Table 2:** Aortic lesion development in F1B Golden-Syrian hamsters fed supplemented non-purified and semi-purified diets.

Ref	Strain	% Fat (w/w)	% CH (w/w)	Diet 1	Diet 2	Outcome	Outcome Variable
[39]	F1B	10^2^	0.1	CO	CLA	↔	CE (ug)/aorta (mg)

[39]	F1B	10^2^	0.1	CO	LA	↔	CE (ug)/aorta (mg)

[39]	F1B	10^2^	0.1	CO	CLA + LA	↔	CE (ug)/aorta (mg)

[105]	F1B	10^2^	0.15	CO	Safflower Oil	↔	CE (ug)/aorta (mg)

[54]	F1B	10^3^	0.1	Palm Oil/CO	Macademia Oil	↔	CE (ug)/aorta (mg)

[54]	F1B	10^3^	0.1	Palm Oil/CO	Canola Oil	↔	CE (ug)/aorta (mg)

[54]	F1B	10^3^	0.1	Palm Oil/CO	Safflower Oil	↔	CE (ug)/aorta (mg)

[54]	F1B	10^3^	0.1	Macademia Oil	Canola Oil	↔	CE (ug)/aorta (mg)

[54]	F1B	10^3^	0.1	Macademia Oil	Safflower Oil	↔	CE (ug)/aorta (mg)

[54]	F1B	10^3^	0.1	Canola Oil	Safflower Oil	↔	CE (ug)/aorta (mg)

[56]	F1B	10^3^	0.1	CO	Olive Oil	↔	CE (ug)/aorta (mg)

[56]	F1B	10^3^	0.1	CO	Safflower Oil	↔	CE (ug)/aorta (mg)

[56]	F1B	10^3^	0.1	Olive Oil	Safflower Oil	↔	CE (ug)/aorta (mg)

[105]	F1B	10^3^	0.15	CO	Safflower Oil	↔	CE (ug)/aorta (mg)

	F1B^1^	10^3^	0.1	CO	Fish Oil	↔	CE (ug)/aorta (mg)

	F1B^1^	10^3^	0.1	Olive Oil	Fish Oil	↔	CE (ug)/aorta (mg)

	F1B^1^	10^3^	0.1	Safflower Oil	Fish Oil	↔	CE (ug)/aorta (mg)

CH: cholesterol; CO: coconut oil; CLA: conjugated linoleic acid; LA: linoleic acid; CE: cholesteryl ester. ^1^Dillard (unpublished data), ^2^Non-purified diet, ^3^Semi-Purified diet

## Conclusion

Early work suggested that hamsters fed diets supplemented with cholesterol and SFA were a useful animal model to study diet induced atherosclerosis. Hamsters appeared to respond to a cholesterol- and SFA-supplemented diet by increasing nHDL-C, ultimately leading to enhanced deposition of cholesterol in the aorta and increased atherosclerotic lesion development. More recent data were less consistent on the usefulness of the model.

The data showed a relatively consistent response of the F1B hamsters fed cholesterol- and SFA-supplemented diets to develop an atherogenic lipoprotein profile (nHDL-C > HDL-C). For those studies in which aortic lesion data were also available, an atherogenic lipoprotein profile did not consistently result in lesion development. No hamster strain consistently developed aortic lesions regardless of atherogenic lipoprotein profile. Data relating other hamster strains to diet induced changes in lipoprotein profile or lesion development were too limited and inconsistent to draw conclusions.

On the basis of limited data, it appears that non-purified diets containing 0.1% to 0.2% (w/w) dietary cholesterol were most commonly associated with eliciting an atherogenic lipoprotein profile without compromising hepatic function. There was insufficient data to determine the effect of dietary cholesterol alone on aortic lesion development. Potential hamster strain differences in response to dietary cholesterol could not be addressed in this review due to insufficient data.

In summary, F1B hamsters fed non-purified diets supplemented with cholesterol and SFA led to a more atherogenic lipoprotein profile compared to all other hamster strains as well as F1B hamsters fed similarly supplemented semi-purified diets. This would suggest that F1B hamsters fed a cholesterol- and SFA-supplemented non-purified diet would be a good model in which to compare and contrast diet-induced changes in plasma lipoprotein concentrations. However, fat type including SFA, MUFA and n-6 PUFA had little effect on plasma lipoprotein concentrations. Cholesterol- and fish oil-supplemented semi-purified diets produced highly variable plasma lipoprotein responses and results antithetical to that observed in humans.

Aortic lesion development in response to dietary fat type was inconsistent and inconclusive because no hamster strain repeatedly developed aortic lesions regardless of background diet, amount of dietary cholesterol, or amount and type of dietary fat.

On the basis of available data it does not appear at this time that the hamster is a useful model to determine the mechanisms underlying the development of diet-induced atherosclerosis. If this model were to be pursued it will be important to develop a standardized atherogenic diet and length of dietary intervention tailored to a specific hamster species, explore potential issues related to sex differences, and come to consensus for the analytical approaches most appropriate to assess the outcome measures of interest.

## List of Abbreviations

Apo: apolipoprotein; CE: cholesteryl ester; CETP: cholesteryl ester transfer protein; CR: Charles River; CVD: cardiovascular disease; HDL-C: high density lipoprotein cholesterol; LDL: low density lipoprotein; MUFA: monounsaturated fatty acids; nHDL-C: non-high density lipoprotein cholesterol; n-3 PUFA: omega-3 polyunsaturated fatty acids; n-6 PUFA: omega-6 polyunsaturated fatty acids; PUFA: polyunsaturated fatty acids; SFA: saturated fatty acids; TG: triglyceride.

## Competing interests

The authors declare that they have no competing interests.

## Authors' contributions

AD, NRM and AHL designed research, AD conducted comprehensive review and analyzed data, AHL provided essential materials, AD and AHL wrote the paper, and AD, NRM and AHL had primary responsibility for final content. All authors read and approved the final manuscript.

## Supplementary Material

Additional file 1 **Plasma lipoprotein concentrations in hamsters fed cholesterol-supplemented non-purified or semi-purified diets**. A set of 4 graphs of plasma lipoprotein concentrations representing studies that fed hamsters cholesterol-supplemented diets without additional fat. Click here for file

Additional file 2 **Plasma lipoprotein concentrations in hamsters fed cholesterol- and fat-supplemented non-purified or semi-purified diets**. A set of 4 graphs of plasma lipoprotein concentrations representing studies that fed different strains of hamsters varying types of fat, including SFA, MUFA, n-6 PUFA and fish oil. Click here for file

Additional file 3 **Aortic lesion development in CR and unknown strains of Golden-Syrian hamsters fed supplemented non-purified diets**. The file contains an additional table of studies that show aortic lesion development in CR and unknown strains of hamsters fed supplemented non-purified diets. Click here for file
