# Supplementary material for: Use of hamster as a model to study diet-induced atherosclerosis
Source: Nutr Metab (Lond). 2010 Dec 10;7:89. doi: 10.1186/1743-7075-7-89 (PMC3004901; doi:10.1186/1743-7075-7-89)
Supplement: Additional file 1 — Plasma lipoprotein concentrations in hamsters fed cholesterol-supplemented non-purified or semi-purified diets. A set of 4 graphs of plasma lipoprotein concentrations representing studies that fed hamsters cholesterol-supplemented diets without additional fat. [file 1743-7075-7-89-S1.DOC]

**
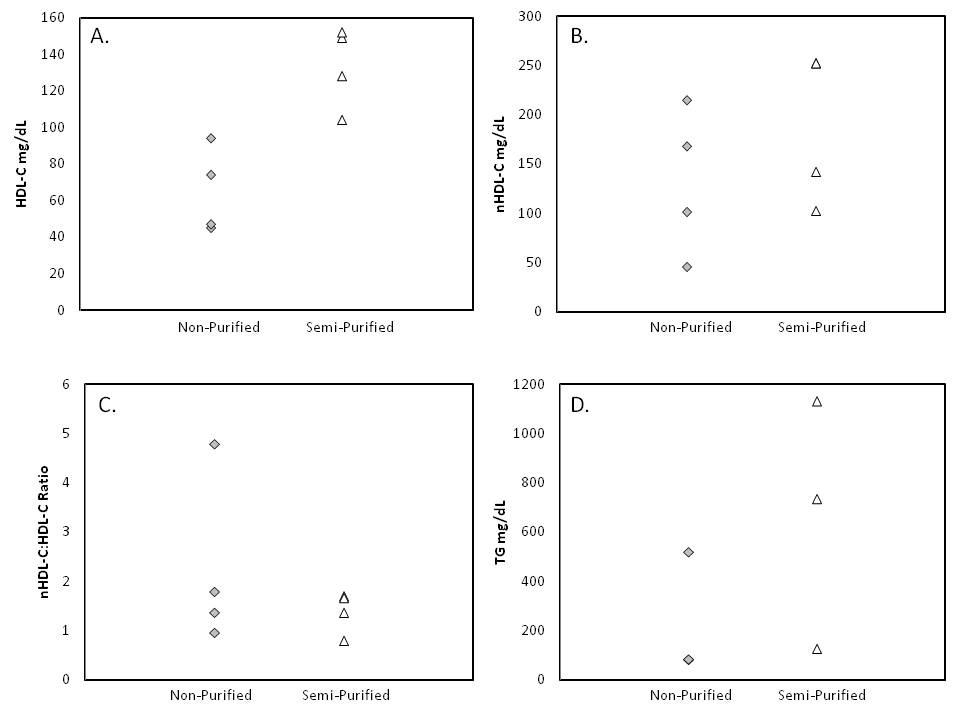
**

**Additional Figure S1. Plasma lipoprotein concentrations in hamsters fed cholesterol-supplemented non-purified or semi-purified diets.** Plasma (A) HDL-C, (B) nHDL, (C) nHDL-C:HDL ratio and (D) TG concentrations in Golden-Syrian hamsters fed cholesterol-supplemented non-purified or semi-purified diets.
