# Supplementary material for: Use of hamster as a model to study diet-induced atherosclerosis
Source: Nutr Metab (Lond). 2010 Dec 10;7:89. doi: 10.1186/1743-7075-7-89 (PMC3004901; doi:10.1186/1743-7075-7-89)
Supplement: Additional file 3 — Aortic lesion development in CR and unknown strains of Golden-Syrian hamsters fed supplemented non-purified diets. The file contains an additional table of studies that show aortic lesion development in CR and unknown strains of hamsters fed supplemented non-purified diets. [file 1743-7075-7-89-S3.DOC]

| **Additional Table S1. CR and unknown strains of Golden-Syrian hamsters fed dietary fat and cholesterol supplemented non-purified diets.** | | | | | | | | |
| --- | --- | --- | --- | --- | --- | --- | --- | --- |
| **Ref** | **Strain** | **% Fat (w/w)** | **% CH (w/w)** | **Diet 1** | **Diet 2** | **Outcome** | **Outcome Variable** |  |
| [47] | CR | 10 | 0.1 | Chow | Canola Oil | ↔ | Aorta with lesion (% ) |  |
| [47] | CR | 10 | 0.1 | Chow | Stick Margarine | ↔ | Aorta with lesion (% ) |  |
| [47] | CR | 10 | 0.1 | Chow | Soybean Oil | ↔ | Aorta with lesion (% ) |  |
| [47] | CR | 10 | 0.1 | CO | Butter | ↔ | Aorta with lesion (% ) |  |
| [47] | CR | 10 | 0.1 | CO | Canola Oil | ↔ | Aorta with lesion (% ) |  |
| [47] | CR | 10 | 0.1 | CO | Soybean Oil | ↔ | Aorta with lesion (% ) |  |
| [47] | CR | 10 | 0.1 | CO | Stick Margarine | ↔ | Aorta with lesion (% ) |  |
| [47] | CR | 10 | 0.1 | Butter | Canola Oil | ↔ | Aorta with lesion (% ) |  |
| [47] | CR | 10 | 0.1 | Butter | Soybean Oil | ↔ | Aorta with lesion (% ) |  |
| [47] | CR | 10 | 0.1 | Butter | Stick Margarine | ↔ | Aorta with lesion (% ) |  |
| [47] | CR | 10 | 0.1 | Canola Oil | Soybean Oil | ↔ | Aorta with lesion (% ) |  |
| [47] | CR | 10 | 0.1 | Canola Oil | Stick Margarine | ↔ | Aorta with lesion (% ) |  |
| [47] | CR | 10 | 0.1 | Stick Margarine | Soybean Oil | ↔ | Aorta with lesion (% ) |  |
| [53] | CR | 10 | 0.1 | Chow + CH | Butter | ↔ | CE (ug)/aorta (mg wet weight) |  |
| [53] | CR | 10 | 0.1 | Chow + CH | Canola Oil | ↔ | CE (ug)/aorta (mg wet weight) |  |
| [53] | CR | 10 | 0.1 | Chow + CH | Stick Margarine | ↔ | CE (ug)/aorta (mg wet weight) |  |
| [53] | CR | 10 | 0.1 | Chow + CH | Soybean Oil | ↔ | CE (ug)/aorta (mg wet weight) |  |
| [53] | CR | 10 | 0.1 | Butter | Canola Oil | ↔ | CE (ug)/aorta (mg wet weight) |  |
| [53] | CR | 10 | 0.1 | Butter | Soybean Oil | ↔ | CE (ug)/aorta (mg wet weight) |  |
| [53] | CR | 10 | 0.1 | Butter | Stick Margarine | ↔ | CE (ug)/aorta (mg wet weight) |  |
| [53] | CR | 10 | 0.1 | Canola Oil | Soybean Oil | ↔ | CE (ug)/aorta (mg wet weight) |  |
| [53] | CR | 10 | 0.1 | Canola Oil | Stick Margarine | ↔ | CE (ug)/aorta (mg wet weight) |  |
| [53] | CR | 10 | 0.1 | Stick Margarine | Soybean Oil | ↔ | CE (ug)/aorta (mg wet weight) |  |
| [48] | CR | 20 | 0.12 | HCO | LA + CLA | ↔ | Aorta with lesion (% ) |  |
| [36] | CR | TAG blend | 0.12 | 8:00 | 18:1 cis | ↑ | Aorta fatty streak area |  |
| [36] | CR | TAG blend | 0.12 | 8:00 | 18:1 trans | ↑ | Aorta fatty streak area |  |
| [36] | CR | TAG blend | 0.12 | 14:00 | 18:1 cis | ↑ | Aorta fatty streak area |  |
| [36] | CR | TAG blend | 0.12 | 14:00 | 18:1 trans | ↑ | Aorta fatty streak area |  |
| [36] | CR | TAG blend | 0.12 | 18:1 cis | 18:1 trans | ↑ | Aorta fatty streak area |  |
| [45] | CR | 10 | 0.4 | OA-Sunflower Oil | Sunflower Oil | ↓ | Aorta fatty streak area |  |
| [37] | CR | 10 | 0.4 | Olive Oil | Sunflower Oil | ↓ | CE (ug)/aorta (mg) |  |
| [64] | UNK | 10 | 0 | CO | Olive Oil | ↑ | Lesion area covered (mm²) |  |
| [57] | UNK | 20 | 0.12 | Butter | Cheese | ↑ | CE (nmol)/aorta |  |

CH: cholesterol; CO: coconut oil; CLA: conjugated linoleic acid; LA: linoleic acid; CE: cholesteryl ester; TAG: triacylglycerol; OA: oleic acid
